# Influence of fast advective flows on pattern formation of *Dictyostelium discoideum*

**DOI:** 10.1371/journal.pone.0194859

**Published:** 2018-03-28

**Authors:** Torsten Eckstein, Estefania Vidal-Henriquez, Albert Bae, Vladimir Zykov, Eberhard Bodenschatz, Azam Gholami

**Affiliations:** 1 Max Planck Institute for Dynamics and Self-Organization, 37077, Göttingen, Germany; 2 Institute for Nonlinear Dynamics, University of Göttingen, 37073, Göttingen, Germany; 3 Laboratory of Atomic and Solid-State Physics and Sibley School of Mechanical and Aerospace Engineering, Cornell University, Ithaca, New York 14853, United States of America; Lanzhou University of Technology, CHINA

## Abstract

We report experimental and numerical results on pattern formation of self-organizing *Dictyostelium discoideum* cells in a microfluidic setup under a constant buffer flow. The external flow advects the signaling molecule cyclic adenosine monophosphate (cAMP) downstream, while the chemotactic cells attached to the solid substrate are not transported with the flow. At high flow velocities, elongated cAMP waves are formed that cover the whole length of the channel and propagate both parallel and perpendicular to the flow direction. While the wave period and transverse propagation velocity are constant, parallel wave velocity and the wave width increase linearly with the imposed flow. We also observe that the acquired wave shape is highly dependent on the wave generation site and the strength of the imposed flow. We compared the wave shape and velocity with numerical simulations performed using a reaction-diffusion model and found excellent agreement. These results are expected to play an important role in understanding the process of pattern formation and aggregation of *D. discoideum* that may experience fluid flows in its natural habitat.

## Introduction

In a reaction-diffusion-advection system one or more reacting species are advected downstream with an externally imposed velocity. This advective flow can induce unique emergent phenomena. An eminent example is the differential flow induced chemical instability (DIFICI) that destabilizes an otherwise spatially homogeneous state of a system [1–3]. The basic idea behind this is that the reacting species flow at different rates. This differential transport can initiate instabilities in an otherwise spatially homogeneous state of the system, leading to propagating wave packets of reactant concentrations traveling in the flow direction. This mechanism of generating spatial structures is free from the constrains of the Turing mechanism [4], which requires a large difference in diffusion coefficients of the two species involved. Accordingly one can expect DIFICI to be found widely in population dynamics [5–8] and biological morphogenesis [9].

The aggregation of *D. discoideum* amoeba after nutrient deprivation is one of the best model systems for the study of spatial–temporal pattern formation at the multicellular level. Upon starvation, *D. discoideum* starts a developmental program as a surviving mechanism. The first part of this process consists of aggregation of 10^4^ − 10^5^ chemotactic cells to form a migrating slug, which then act as a multicellular organism to search for nutrients. Because of this, *D. discoideum* has been largely studied to understand the transition from uni- towards multicellularity. The aggregation of amoebas is achieved by using the signaling chemical cyclic adenosine monophosphate (cAMP), which is initially secreted by some of the amoebas and then relayed by the others. The patterns produced by cAMP have attracted a lot of attention in the pattern formation community, since they are a primary example of spiral waves and target patterns in nature. Regarding spiral waves and target patterns in *D. discoideum* see for example Refs. [10–13]. These structures then constitute the centers to which the amoebas aggregate. However, in their natural habitat in the forest soil, *D. discoideum* cells are subjected to flows which advect cAMP, thus affecting the signaling process. In soils, rainwater speeds can vary from values near zero up to around 250 mm/min, which is one order of magnitude larger than flow rates studied in this work [14]. It is not yet clear how these advective flows affect the aggregation of *D. discoideum* cells in nature.

Recently, we have conducted experiments and performed numerical simulations to study flow-driven waves in a biological system, namely quasi one-dimensional colonies of signaling amoeba *D. discoideum* [15]. In these experiments with chemotactically competent *D. discoideum* cells, a straight flow-through microfluidic channel was used. Starved cells were allowed to settle on the substrate before a laminar flow of buffer was switched on. The flow advected extracellular cAMP downstream but was not strong enough to detach the cells from the substrate. This differential transport of extracellular cAMP induced macroscopic wave trains that had a unique period and propagated with a velocity proportional to the imposed flow velocity downstream. This behavior was studied theoretically [16, 17] using the two-component reaction-diffusion model proposed by Martiel-Goldbeter [18] for the production and relay of cAMP. While the theoretical results could explain much of the experimental observations, there were still open questions regarding the generation of a self supporting wave train at the inlet of the microfluidic channel and only small flow rates of up to 5 mm/min were studied. Furthermore, the state of the cells was assumed to be constant in the convectively unstable regime, lacking a way to verify this experimentally.

In this work, we extend our experiments to investigate flow-driven waves at high flow rates in the same microfluidic set up. The flows are not yet strong enough to detach the cells from the substrate. We observed elongated waves that extend over the whole length of the channel and propagate both perpendicular and parallel to the flow direction. We characterized the wave shape as well as the wave propagation velocity and compared them to the numerical simulations of the system. Moreover, we changed the imposed flow rates abruptly to study the system response both experimentally and by means of numerical simulations. In our comprehensive numerical study, we found that the two-component Martiel-Goldbeter model does not correctly reproduce the wave shape observed experimentally at a higher flow velocities. However, a three-component approach successfully reproduces the wave shape while still matching with the experimental results in period and wave speed. Additionally, we found that sustained wave formation can be induced using a developmental path model [19] for the state of the cells in the channel. This seems a very reasonable assumption, since *D. discoideum* cells change the activity of a number of genes during the aggregation process [20]. We could also reproduce the experimental observations for rapid flow switching by assuming a mixture of oscillatory and excitable cells.

## Materials and methods

### Cell culture

All experiments were performed with *D. discoideum* AX2-214 cells, kindly provided by Günther Gerisch (MPI for Bio-chemistry, Martinsried, Germany). Cells were grown in HL-5 medium (35.5*g* of Formedium powder from Formedium Ltd, England, per liter of double-distilled water, autoclaved and filtered) at 22°C on polystyrene Petri dishes (TC Dish 100, Sarsted, Germany) and harvested when they became confluent. Before the experiments, the cells were centrifuged and washed two times with phosphate buffer (2*g* of KH_2_PO_4_ and 0.36*g* of Na_2_HPO_4_.H_2_O per liter at pH 6.0, autoclaved, both from Merck, Germany). The centrifuged cells were resuspended in 10 ml of the same buffer and transferred into a shaking Erlenmeyer flask (150 rpm) for starvation. After approximately one hour, the cells were centrifuged at 1000 rpm for 3 min and resuspended in 200 *μ*l fresh phosphate buffer. The cell density was determined using a hemocytometer (Neubauer Zählkammer), diluted to 5 × 10^7^ cells/ml of phosphate buffer and filled into the microfluidic channel.

### Microfluidics

The microfluidic devices were fabricated by standard soft lithography [21]. A silicon wafer was coated with a 100 *μ*m photoresist layer (SU-8 100, Micro Resist Technology GmbH, Berlin, Germany) and patterned by photolithography to obtain a structured master wafer. The channels are 2 mm wide, 50 mm long, and 103 ± 2 *μ*m high. Polydimethylsiloxane (PDMS, 10:1 mixture with curing agent, Sylgard 184, Dow Corning GmbH, Wiesbaden, Germany) was poured onto the wafer and cured for 2 h at 75° C. To produce the microfluidic device, a PDMS block containing the macro-channels was cut out, and two inlets (7 mm and 0.75 mm in diameter) were punched through the PDMS at opposite ends of the channel with the help of PDMS punchers (Harris Uni-Core-7.00 and Harris Uni-Core-0.75). Afterwards, a glass microscope slide (76×26 mm, VWR) was sealed to the PDMS block following a 20–30 s treatment in air plasma (PDC 002, Harrick Plasma, Ithaca, USA) to close the macro-channels. The large inlet was used as a liquid reservoir and from the other side phosphate buffer was pumped out using a high precision syringe pump (PHD 2000 Infuse/Withdraw Syringe Pump from Harvard Apparatus, USA, combined with gasstight glass syringes from Hamilton, USA) at constant buffer flow rate. Moreover, given the dimension of the channel and the dynamics viscosity of the flowing phosphate buffer (*η* = 10^−3^ Pa s), one can calculate the shear stress applied on the cells at the highest imposed flow velocity of *V*_*f*_ = 50 mm/min to be *σ* = 0.046 Pa (see supplementary S1 File). According to the literature, mechanosensing in *D. discoideum* has been observed above a threshold of *σ* = 0.7 Pa, and cell detachment from substrate occur at higher threshold of *σ* = 2.7 Pa [22]. We are thus one order of magnitude below the regime where flow induced shear stress would bias the motion of chemotactic cells or even detach the cells from substrate.

### Image acquisition and analysis

We used a dark-field setup consisting of a monochrome 12-bit CCD camera (QIClick-F-M-12 from QImaging), a 50 mm focal length objective (MVL50M23 from Thor Labs), a 7 inch focal length fresnel lens (11.0” x 11.0”, 7” Focal Length from Edmund Optics, bottom side in-house coated with an anti-reflective coating) and a ring of green LEDs as light source (LED Miniatur Ringbeleuchtung LSR24-G from LUMIMAX). The camera was controlled with an image capture program (Micro-Manager [23]) and recorded images every 20 seconds. To process dark-field images, we first subtract them from each other (image number *n* from image number *n*+3) [24] and then band-passed filtered where large structures are filtered down to 3.5 mm and small structures up to 0.294 mm. Finally, to calculate the phase map, at each pixel we first subtracted the time average of the signal and then performed the Hilbert transform [25].

### Numerical simulations

**Fig 1:**
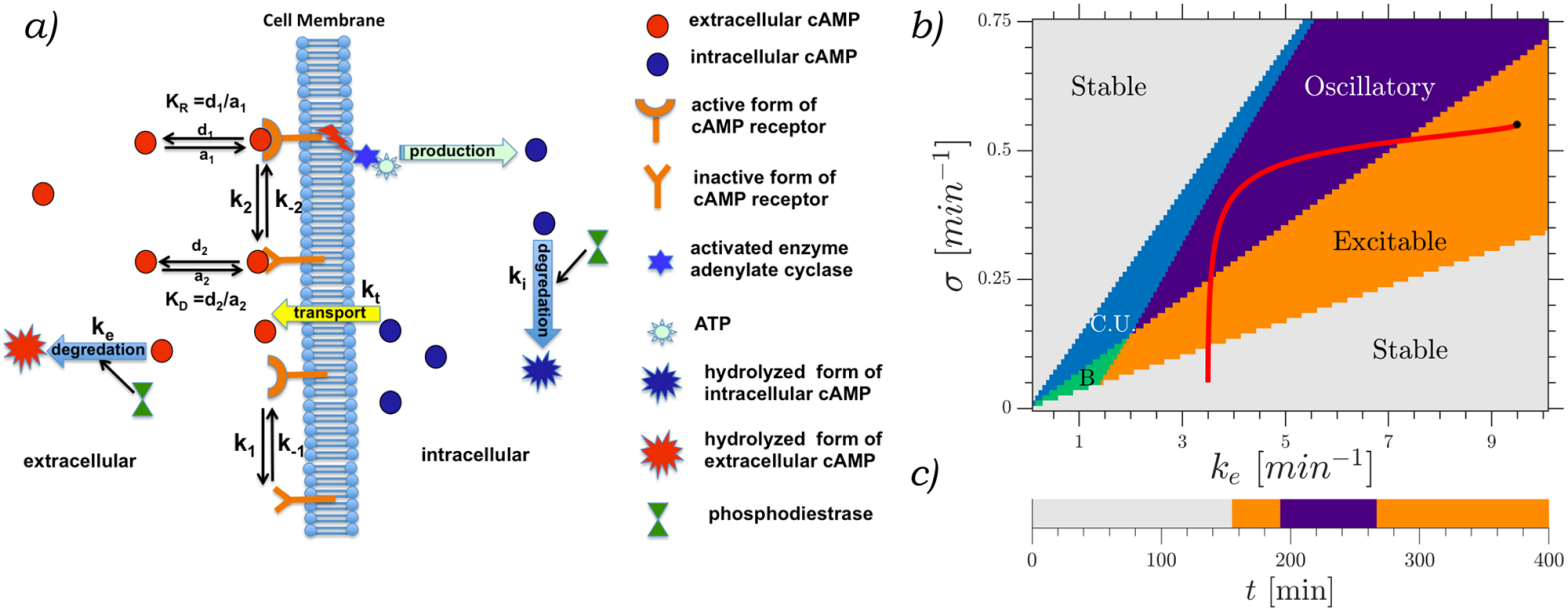
a) Schematic representation of the reaction-diffusion model used, reproduced from [16]. b) Phase diagram showing the different regimes depending on the production *σ* and degradation *k*_*e*_. Stable regime in white, where one stable steady state exists, excitable regime in orange, 3 steady states, one of which is excitable and the other two unstable. Oscillatory regime in purple, one unstable steady state surrounded by a limit cycle. Convectively unstable regime in light blue, one steady state which is convectively unstable. Bistable regime in green, two stable steady states. The red line marks the trajectory that the developmental path follows. Simulations with fixed parameters used the ones marked by the black asterisk. c) Cell state over time for a cell starting with *t*_*s*_ = 0. The color coding is the same as b).

**Fig 2:**
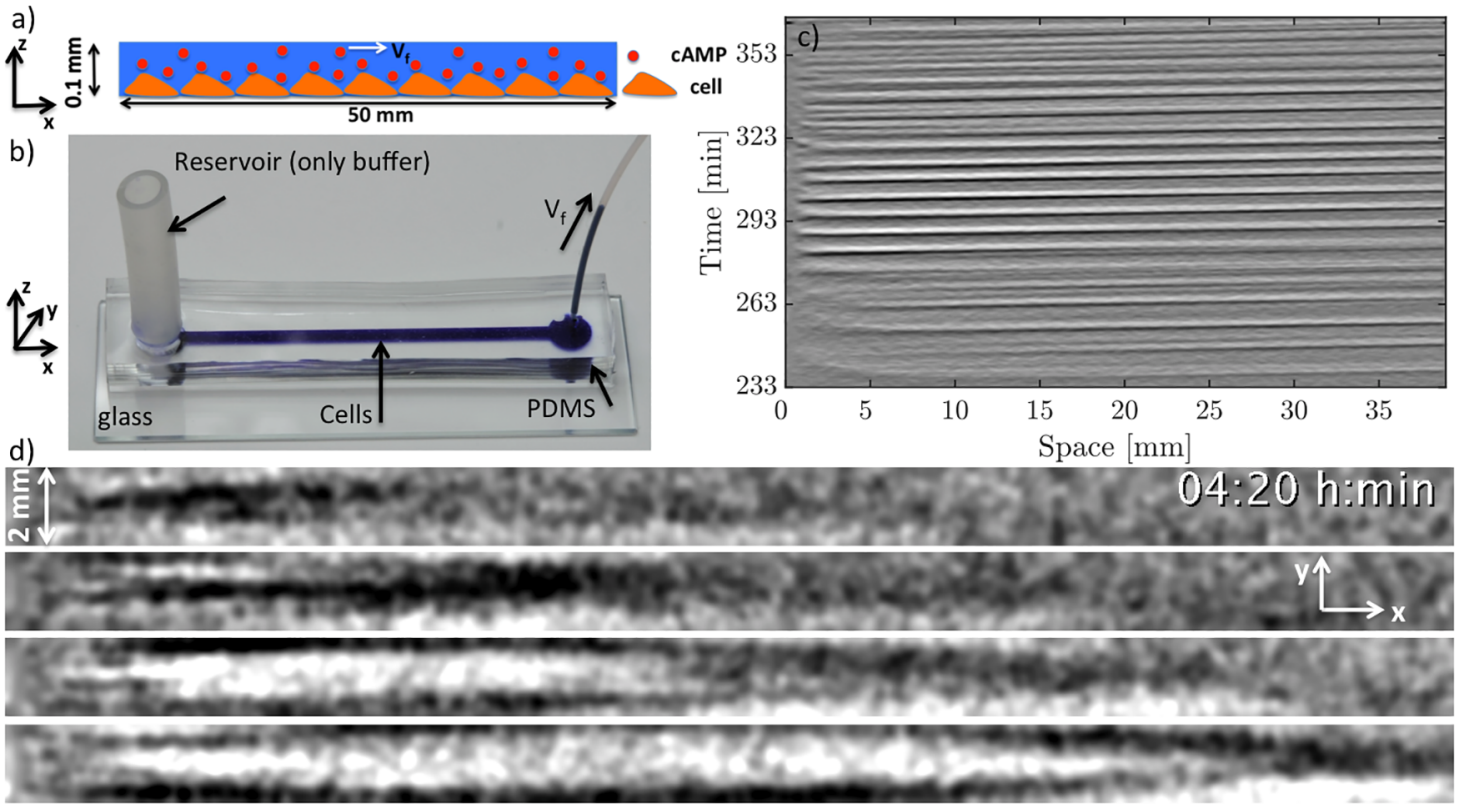
a) A schematic side view of the channel loaded with cells that are attached to the substrate and exposed to an external fluid flow advecting cAMP molecules downstream. b) Experimental setup filled with blue ink for better visualization. The reservoir is filled only with buffer and the liquid is pumped out with a syringe pump from the right side. c) Space-time plot of the flow-driven waves at the imposed flow velocity of *V*_*f*_ = 10 mm/min. d) Snapshots of the waves taken from the top of the channel obtained by subtracting successive images (captured every 20 sec) of the channel every 1 min (image number *n*+3 minus image number *n*) and bandpass filtered. The time increment between successive images is 1 min. Time stamp shows the time since the start of starvation.

We conducted numerical simulations of the model proposed by Martiel and Goldbeter [18] for the production and relay of cAMP, with the addition of an advection term to account for the imposed flow (see Fig 1a). The reaction-diffusion set of equations model (in 2-D) the amount of cAMP in the extracellular medium *γ*(*x*, *y*), the amount of cAMP in the intracellular medium *β*(*x*, *y*), and the percentage of active receptors on the outside of the cell membrane *ρ*(*x*, *y*), where *x*, *y* are cartesian spatial coordinates. This last field *ρ*(*x*, *y*) quantifies the affinity of the cell receptors to bind with cAMP, thus providing the refractory time for this excitable medium. We use a 2-D approximation of the shape of the channel (*x* − *y* plane in Fig 2b) due to its low aspect ratio in *z* direction. The equations are as follow

$$\partial_{t}\rho =k_{1}[-f_{1}\left(\gamma \right)\rho +f_{2}\left(\gamma \right)\left(1-\rho \right)],$$ (1a)

$$\partial_{t}\beta =q\sigma \alpha \Phi \left(\rho ,\gamma \right)/\left(1+\alpha \right)-\left(k_{i}+k_{t}\right)\beta ,$$ (1b)

$$\partial_{t}\gamma =D\nabla^{2}\gamma -v\left(y\right)\cdot \nabla \gamma +k_{t}\beta /h-k_{e}\gamma ,$$ (1c)

with

$$\begin{array}{c} f_{1}\left(\gamma \right)=\frac{1+\kappa \gamma }{1+\gamma },\;f_{2}\left(\gamma \right)=\frac{L_{1}+\kappa L_{2}c\gamma }{1+c\gamma },\;\Phi \left(\rho ,\gamma \right)=\frac{\lambda_{1}+Y^{2}}{\lambda_{2}+Y^{2}},\;Y\left(\gamma ,\rho \right)=\frac{\rho \gamma }{1+\gamma }, \end{array}$$

$\nabla =\partial_{x}\hat{x}+\partial_{y}\hat{y}$, and $v\left(y\right)=v\left(y\right)\hat{x}$. Eq 1a models the process of desensitization and recovery of the active receptors given by *f*_1_ and *f*_2_, respectively. Eq 1b characterizes the changes in cAMP inside the cells given by the nonlinear production term Φ. The amount of intracellular cAMP is reduced by intracellular degradation and transport to the extracellular medium at rates described by *k*_*i*_ and *k*_*t*_, respectively. Finally, Eq 1c represents the changes on *γ* given by degradation through phosphodiesterase at a rate *k*_*e*_ and the transport from the intracellular medium at a rate *k*_*t*_. These processes are schematically represented in Fig 1a. *γ* is subjected to diffusion and advection, the other two fields do not diffuse nor advect since they are attached to the cells. For a detailed derivation of this model please refer to the original works of Martiel and Goldbeter [18] and Tyson et. al. [26]. The parameters used are *k*_1_ = 0.09 min^-1^, *κ* = 18.5, $L_{1}=10$, $L_{2}=0.005$, *c* = 10, *q* = 4000, *α* = 3, λ_1_ = 10^−4^, λ_2_ = 0.2575, *k*_*i*_ = 1.7 min^-1^, *k*_*t*_ = 0.9 min^-1^, *D* = 0.024 mm^2^/min, *h* = 5.

We simulated this system using a Runge-Kutta scheme with a Merson error aproximation [27] to ensure numerical accuracy. Nonlinear discretization was used for the advection operator in order to deal with high velocities while keeping a non-negative concentration of cAMP [28]. We used a no flux (∂_*x*_*γ*(*x* = 0) = 0) boundary condition in all boundaries, including upstream, and kept the same parameters as in our previous simulations [29] while keeping freedom to move in the parameter space characterized by *σ* and *k*_*e*_. We conducted simulations both with fixed parameters (over time and space) and with a developmental path based on the work by Laurenzal et al [19]. When we used this path, the parameters *k*_*e*_ and *σ* were changed from being uniform in the whole system to being particular to each cell group (patch). Each patch had an area of 0.1 mm × 0.1 mm and a particular starting time along the cellular developmental path. This path takes the cells from having one stable solution, to an excitable regime, one oscillatory solution, and then back to excitable (see Fig 1b for an overview of the different regimes in this system), by changing with time the parameters *σ* and *k*_*e*_ according to

$$\begin{array}{c} \sigma \left(t\right)=0.3+0.25\tanh(\frac{t+t_{s}-200}{50}),\;k_{e}\left(t\right)=6.5+3\tanh(\frac{t+t_{s}-260}{30}), \end{array}$$

where *t*_*s*_ corresponds to the initial development time of a patch and *t* is the simulation time. The starting times were selected following an exponential distribution with a rate parameter Δ^−1^,

$$\begin{array}{c} P\left(t_{s}\right)=\frac{e^{-t_{s}/\Delta }}{\Delta } \end{array}$$

In all our simulations Δ = 25 min. The advection velocity *V*_*f*_ was selected to be constant along the longest axis of the channel (*x*-axis) while the *y*−axis dependency was calculated using the Navier-Stokes equation with the assumption of a laminar Pouseuille flow. This gives a flow that is mostly planar with a sharp drop at the boundaries, with a boundary layer of about 50 *μ*m, which is of the order of half the height of the channel (see supplementary information). The system was initiated with each patch at its steady state. Different initial states were tested and did not seem to influence the final results, since the system quickly relaxes to its steady state.

We also performed simulations using the two-component version of this model, which makes the assumption that the intracellular production of cAMP is immediately transported to the extracellular medium. This is achieved numerically by setting ∂_*t*_*β* = 0, thus the set of equations becomes

$$\partial_{t}\gamma =D\nabla^{2}\gamma -v\left(y\right)\cdot \nabla \gamma +s\Phi \left(\rho ,\gamma \right)-k_{e}\gamma ,$$ (2a)

$$\partial_{t}\rho =k_{1}[-f_{1}\left(\gamma \right)\rho +f_{2}\left(\gamma \right)\left(1-\rho \right)],$$ (2b)

where *s* = *qk*_*t*_ *ασ*/(*h*(*k*_*t*_ + *k*_*i*_)(1 + *α*)). All parameters used are the same as in Eq 1.

## Results

### Characterization of the flow-driven waves at high flow rates

**Fig 3:**
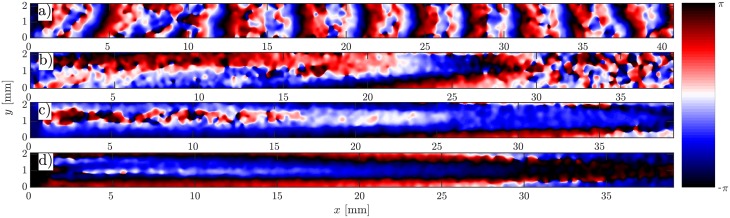
Phase map of the flow-driven waves showing different wave shapes at different imposed flow velocities. a) Practically planar wave fronts at *V*_*f*_ = 0.5 mm/min. b) Parabolic shape at *V*_*f*_ = 5 mm/min. Extremely elongated parabolic wave fronts at flow velocities of *V*_*f*_ = 10 mm/min and *V*_*f*_ = 15 mm/min are shown in c) and d), respectively.

**Fig 4:**
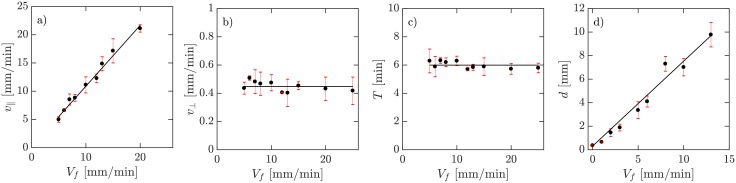
Experimental data on dependency of a) wave speed along the channel *v*_∥_, b) wave speed in transversal direction *v*_⊥_, c) wave period *T*, and d) wave front thickness as a function of imposed flow velocity *V*_*f*_. Continuous lines represent in a), d) least square fit assuming linear scaling and in b), c) average transversal propagation velocity and wave period.

In the absence of flow, signaling *D. discoideum* cells synchronize and show formation and propagation of spiral waves (see supplemental S1 Video). When subjected to advective flows, the spiral patterns are replaced by wave trains traveling downstream. Fig 2d shows an example of flow-driven waves for an average flow velocity of *V*_*f*_ = 10 mm/min. The image contrast reflects the shape changes of the cells. The light bands correspond to high concentrations of cAMP and consist of elongated cells while in the dark bands the cAMP concentrations is small and cells remain round [12, 30–32].The corresponding space-time plot is shown in Fig 2c, where light intensity is averaged over the 2 mm width of the channel and then stacked up along the time axis. The slope of the diagonal bands give the inverse of the average propagation velocity of the waves along the channel. The wave shape and propagation speed strongly depend on the strength of the imposed flow velocity. At small flow rates, a wave train develops spontaneously that fills the whole length of the channel (Fig 3a and supplementary S2 Video). The wavelength of the traveling waves increases linearly with the imposed flow velocity [15] and becomes comparable or larger than the length of the microfluidic channel at high flow rates (Fig 3b–3d). Deformations of the wave front also increase significantly with the imposed flow velocity. Planar wave fronts at small flow rates deform to parabolic fronts at intermediate velocities and become extremely extended at higher flow speeds (Fig 3b–3d, and supplementary S3, S4 and S5 Videos). Notice that the wave shape does not reflect the flow profile which is relatively constant across the width of the channel and drops quickly to zero at a length scale comparable to half of the channel’s height (50 *μ*m), see supplementary S1 Fig and Ref. [29] for detailed calculations of the flow profile. Moreover, at small and intermediate flow rates, the waves propagate solely in the flow direction. However, at higher flow rates they propagate both in the flow direction as well as transversal to the imposed flow. While propagation speed along the flow (*v*_∥_) is comparable to the imposed flow velocity, the transversal propagation speed (*v*_⊥_) is much smaller and of the order of the propagation velocity of waves emitted by spirals in this system in the absence of flow (*v*_⊥,*avg*_ = 0.45 ± 0.04 mm/min). The wave period *T* shows no clear velocity dependence, and takes on a value of *T*_*avg*_ = 5.98 ± 0.25 min. We also measured the width of the wave fronts *d*, as a function of the imposed flow velocity. We found a linear dependency which is shown in Fig 4d. For this measurement, we calculated the second moment of the light intensity *I* at the middle of the channel defined as $\sigma^{2}=\sum_{i}I_{i}{\left(x_{i}-\bar{x}\right)}^{2}/\sum I_{i}$, and multiplied *σ* by the factor of 2.355 to obtain *d* as the “full width at half maximum” (FWHM) of a Gaussian distribution with standard deviation *σ*.

**Fig 5:**
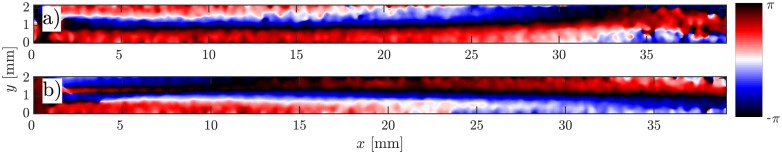
a) A half-parabolic shaped wave front observed at *V*_*f*_ = 10 mm/min. b) Two stripe-like waves initiating at top and bottom boundaries for flow speed of *V*_*f*_ = 15 mm/min.

At high flow velocities, the wave generation site plays an important role for its final shape. If the wave is initiated close to the vertical middle of the channel, it propagates along the length and across the width of the channel. Since the wave propagation velocity parallel to the flow is much faster than perpendicular to it, the wave gets stretched along the channel. This leads to the formation of an elongated parabolic-shaped wave front (Fig 3c and supplemental S4 Video). However, if the initial excitation is in the vicinity of top (*y* = 2 mm) or bottom boundaries (*y* = 0), the wave can only propagate in one direction across the channel, which results in a half-parabola wave front, as shown in Fig 5a and supplemental S4 Video. At very high speeds (*V*_*f*_ ≥ 15 mm/min), we observe an extreme version of this process where stripe-like patterns form, as shown exemplary in Fig 5b for *V*_*f*_ = 15 mm/min and supplemental S5 Video.

### Numerical simulations results

**Fig 6:**
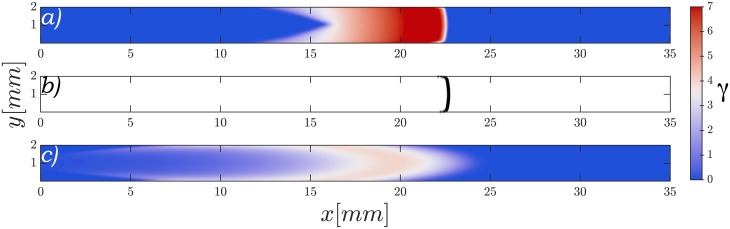
Comparison of wave shapes. For a) the two-component model and c) the three-component model an initial perturbation was applied center upstream on the channel and advected at *V*_*f*_ = 10 mm/min. Panel b) shows a group of particles with no interaction between them starting at the same position as the perturbation in a) and being advected at *V*_*f*_ = *v*_⊥_+10 mm/min, with *v*_⊥_ = 1.8 mm/min the velocity of the two-component model wave without advection.

To study the wave shape in our system in a more detailed manner, we performed numerical simulations of the two-component model (Eq 2) at high flow speeds and fixed parameters *σ* = 0.55 min^−1^, *k*_*e*_ = 9.5 min^−1^ (excitable regime). Starting with an initial perturbation centered upstream in the channel, we observed that the produced waves do not acquired a parabolic shape, but rather a planar form very similar to the flow profile applied as it is shown in Fig 6a and supplementary S6 Video. We compared these patterns to simulations of inert particles being advected with the same flow, and found very good agreement between the two as shown in the top two panels of Fig 6. This direct correspondence between the wavefront evolution and the advection velocity is due to the instantaneous reaction of the cells to the extracellular presence of cAMP.

**Fig 7:**
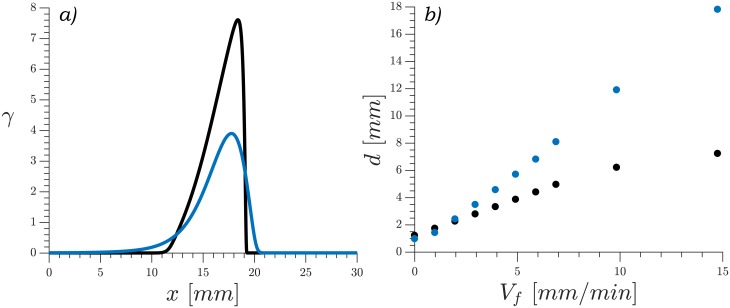
a) Wave profile comparison between the two- and three-component models at imposed flow velocity of *V*_*f*_ = 5 mm/min. b) Wave thickness vs imposed flow for the different models; calculated as 2.355 times the square root of the second moment. Two-component model in black, three-component model in blue.

In contrast to the two-component model, simulations of the three-component model (Eq 1) with the same parameters gave a shape much more similar to the experiments as can be seen in Fig 6c and supplementary S7 Video. In this version of the model there is a non-instantaneous transport of cAMP between the intracellular and the extracellular media, thus slowing down the waves and effectively shrinking the difference between the velocities across and along the channel, allowing for more rounded shapes. The striking difference between the waves generated by the two models can also be appreciated in the wave profile under advective flow shown in Fig 7a. Here it can be seen that in the fast dynamics model the front of the wave is very sharp, with the cAMP rising to its maximum value very quickly. In the three-component model the wave build up is much slower showing a softer curve that looks more similar to our experimental observations.

We found the velocity of the observed waves to increase linearly with the applied flow for both models, in agreement with the experiments. We also observed an increase of the thickness on the wave profile with increased advection flow. To characterize this, we calculated 2.355 *σ*, where *σ*^2^ is the second moment of the wave along the middle of the channel defined as $\sigma^{2}=\sum \gamma_{i}{\left(x_{i}-\bar{x}\right)}^{2}/\sum \gamma_{i}$. These results are shown in Fig 7b. The increase is faster in the three-component model than in the two-component one, consistent with the profile shown in Fig 7a.

**Fig 8:**
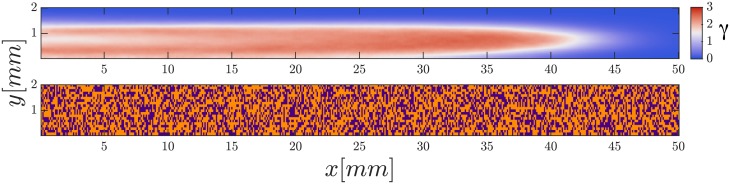
Parabolic-shaped wave observed in simulations using a developmental path. Advecting flow *V*_*f*_ = 15 mm/min. Top: cAMP concentration. Bottom: State of the cells at the moment of wave initiation: Excitable cells in orange and oscillatory cells in purple. The wave is initiated upstream almost at the middle of the channel.

**Fig 9:**
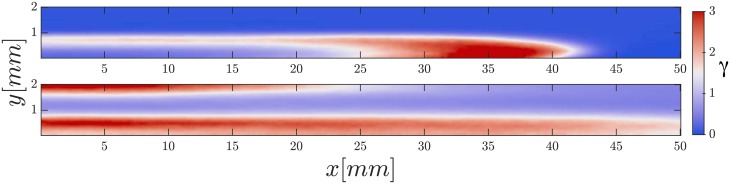
Elongated waves observed in simulations using a developmental path. Advecting flow *V*_*f*_ = 15 mm/min. Top and bottom panels show cAMP waves in simulations with two different initial conditions in the state of the cells.

Finally, we performed simulations with cells following a developmental path as described in Materials and Methods. Similarly as previously observed in [33], we see cAMP waves starting from cells more advanced in their developmental path, i.e. higher *t*_*s*_. When we tried varying the patch size we observed that a minimum amount of cells together in the oscillatory regime were necessary to initiate a wave. For bigger patches, one patch was enough to initiate a wave. Interestingly, we observed that at high speeds (above 2 mm/min) only oscillatory patches at the left end (upstream) of the channel generate waves. Advanced cell clusters down the channel failed to produce waves. The wave shapes observed were of a wide variety, very similar to the ones observed in experiments. The numerical waves presented in Figs 8 and 9 can be compared to the experimental ones of Fig 3, showing very elongated parabolic-shape waves and waves moving perpendicularly to the flow (see supplementary S8 and S9 Videos).

### On- and off- cycles of the imposed flow

To verify our assumptions on the dynamical state of the cells in the numerical simulations, we performed experiments in which we abruptly switched off the imposed flow, after the flow-driven waves had been fully established throughout the channel. This lets us to distinguish between real waves of cAMP and phase waves, as both types of waves respond differently to changes in flow rate. If the cells are mostly in the oscillatory regime, we expect the waves to be phase waves. Since a phase wave is not directly induced by the diffusing chemicals, it should travel at the same velocity and width after turning off the flow. In contrast, an excitation (trigger) wave should propagate at the normal speed selected nonlinearly by the reaction-diffusion balance of the system and should also recover its standard width in the absence of the flow. Finally, it is also possible that the waves are not stable under abrupt changes of the flow rate.

**Fig 10:**
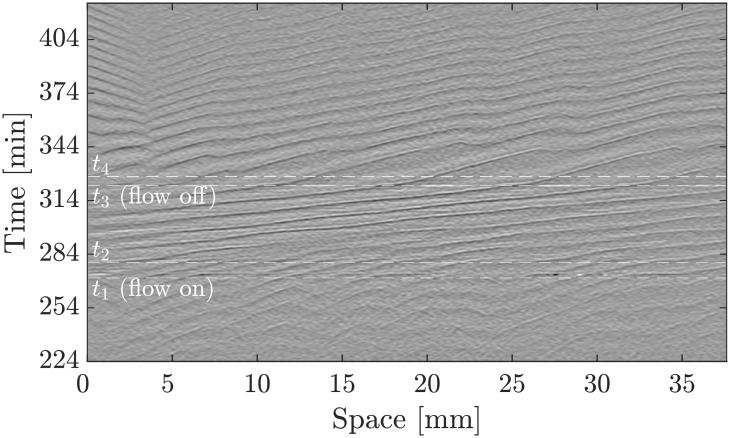
Space-time plot of an experiment in which the flow was initially absent, then turned on (*V*_*f*_ = 1 mm/min) at *t*_1_ and turned off again at *t*_3_. While the flow is off (*t* ≤ *t*_1_), the cells show target patterns. After it turns on at *t*_1_, there is a short disordered phase until flow-driven waves fully develop, which travel downstream at *v*_∥,*on*_ = 0.99 ± 0.03 mm/min. At time *t*_3_, the flow is turned off and the waves still propagate further downstream at slower speed of *v*_∥,*off*_ = 0.37 ± 0.03 mm/min for 30 min. They ultimately vanish on collision with waves emitted from new centers.

**Fig 11:**
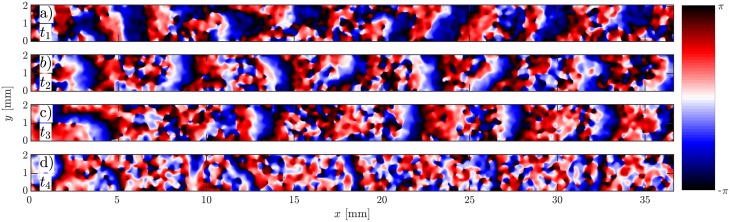
a) The wave pattern at the time point that the flow of magnitude *V*_*f*_ = 1 mm/min is turned on (*t*_1_ in Fig 10), and b) at time *t*_2_, 8 min later. c) The fully developed waves shortly before turning off the flow at time *t*_3_ and d) the waves at time *t*_4_, shortly after switching off the flow.

Thus, we performed experiments in which we switched off the imposed flow while there were flow-driven waves clearly visible in the channel. The corresponding space-time plot of this experiment is shown in Fig 10. We find that in the presence of an external flow, the waves have a higher amplitude as it can be seen in Fig 10 and supplementary S10 Video. The thickness of the wave fronts becomes two to three times larger in the presence of flow (see Fig 11). For a number of experiments, we observed that the waves in the channel would slow down and travel further along the channel with their typical velocity of 0.4 mm/min in the absence of advection, as shown in Fig 11 and S10 Video. However, these waves usually did not traverse the channel very far, being annihilated by emitted waves from newly formed centers. These observations confirm that these propagating waves are trigger waves and at least a portion of the cells are in the excitable regime.

**Fig 12:**
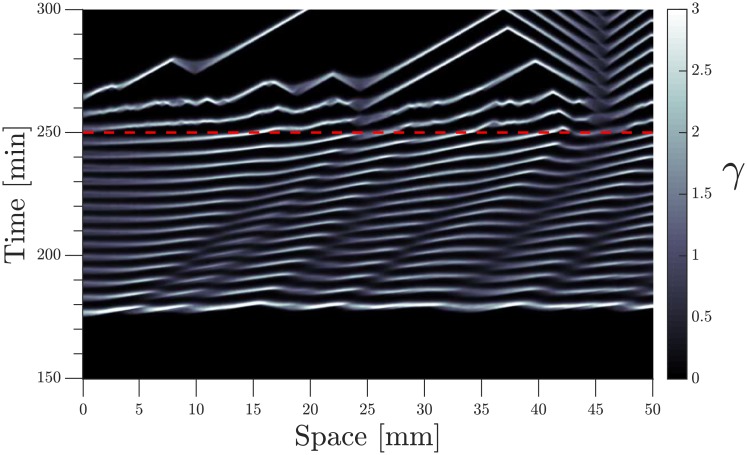
Space-time plot of numerical simulations using the developmental path scheme. The advecting flow is initially *V*_*f*_ = 1 mm/min and stops at *t* = 250 min.

We also performed numerical simulations with a similar setup, that is, with a developmental path scheme and switching off the flow once the waves were formed. Results from those simulations are presented as a space-time plot in Fig 12 and supplementary S11 Video. We observed a change in wave thickness and velocity once the flow is switch off, with some waves continuing traveling at a smaller speed, showing good agreement with the experimental observations.

**Fig 13:**
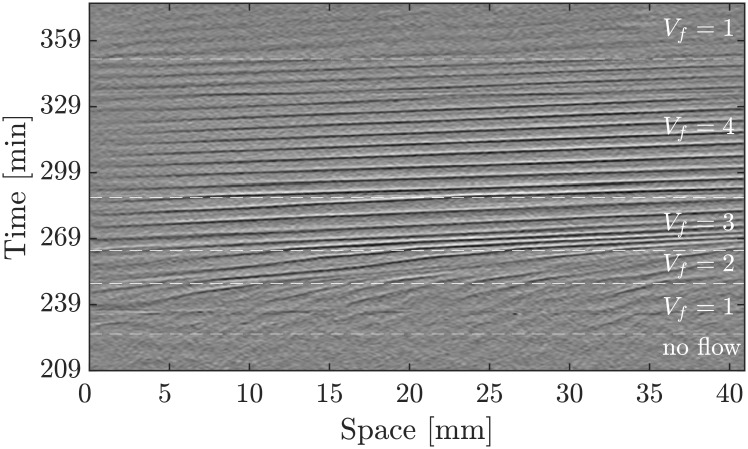
Propagating waves follow the velocity jumps of the imposed flow from 1 mm/min to 4 mm/min. The waves already established at *V*_*f*_ = 2 mm/min accelerate as the flow increases to 3 mm/min, and to keep the wavelength already set at *V*_*f*_ = 2 mm/min, period decreases transiently for these waves to 4 min.

Finally, we increased step-wise the imposed flow velocity to further study the system response. Fig 13 shows the space-time plot of an experiment where the imposed flow increases from 1 mm/min to 4 mm/min and returns back to 1 mm/min at the end. The slope of the diagonal bands, which give a measure of the inverse wave velocity, follow the velocity jumps of the applied flow (see supplementary S12 Video). Interestingly, we observe a transient decrease in the wave period as the imposed velocity changes from 2 mm/min to 3 mm/min. Since the wavelength is already fixed for the previously developed waves at 2 mm/min, they adjust to higher speed by decreasing the period to 4 min (roughly 2/3 of the normal 6 min period). Newly developed waves at the inlet area of the channel (*V*_*f*_ = 3 mm/min), have a higher wavelength and velocity, and the wave period recovers back to the standard value of 6 min.

**Fig 14:**
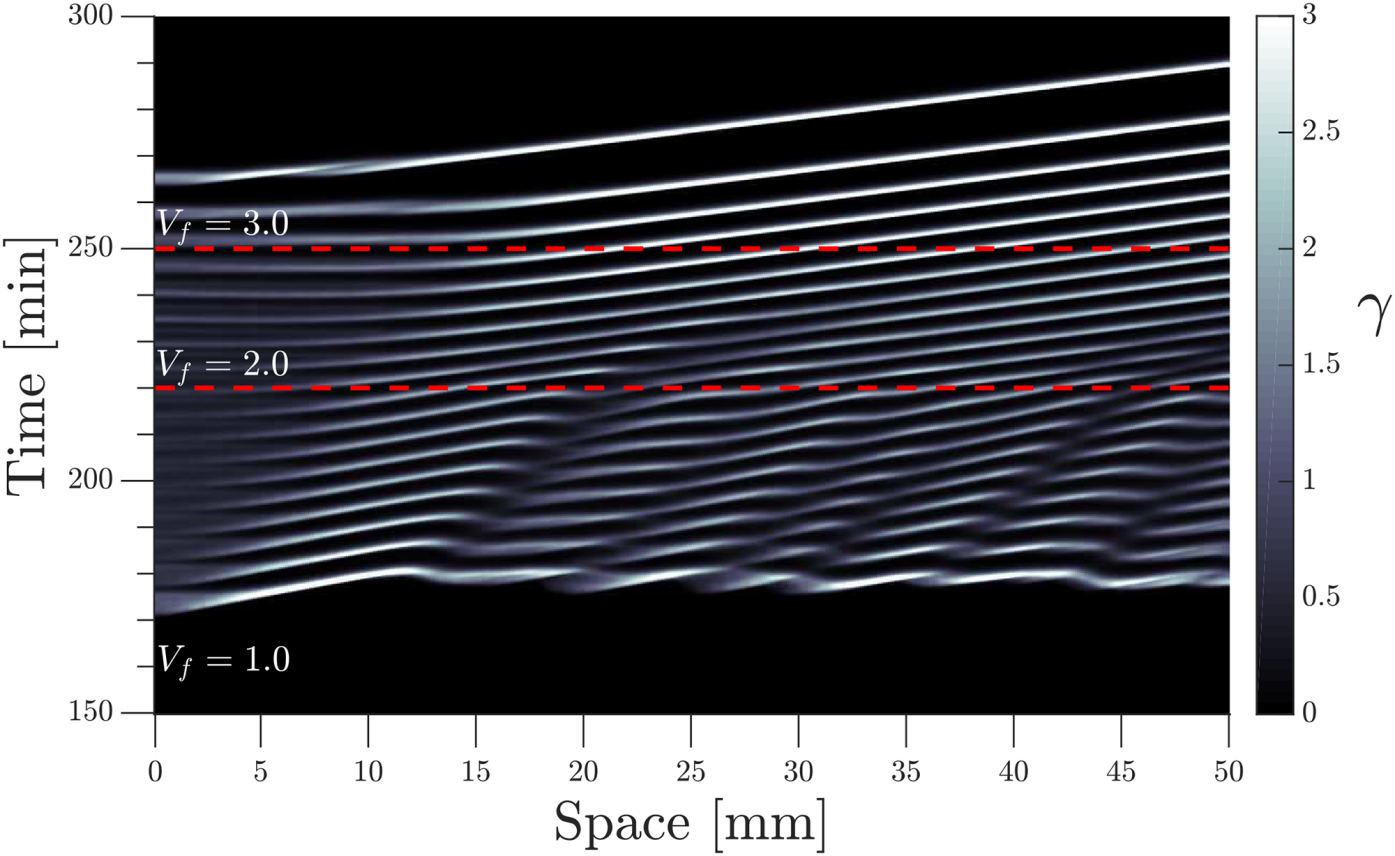
Space-time plot of numerical simulations using the developmental path scheme and a stepwise incremental flow. Initial flow velocity *V*_*f*_ = 1.0 mm/min, incremented at *t* = 220 min to *V*_*f*_ = 2.0 mm/min, and again increased to *V*_*f*_ = 3.0 mm/min at *t* = 250 min.

Numerical simulations of a similar system with developmental path scheme is shown in Fig 14 and supplementary S13 Video, where the flow velocity is increased stepwise from 1 mm/min to 3 mm/min.

### Aggregation under the influence of flow

**Fig 15:**
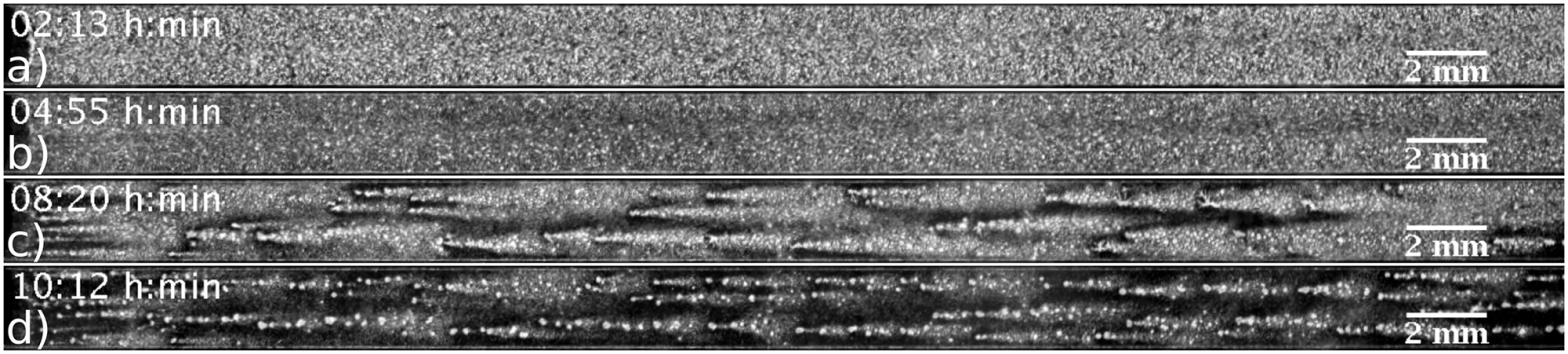
a) Uniform cell distribution at the beginning of experiment in a flow-through microfluidic channel (*V*_*f*_ = 10 mm/min). b) During the propagation of the waves, the variations in cell density due to chemotactic cell movement are still negligible. c) Aggregation patterns after 8 hours starvation show cone-shaped structures with long streams downstream of the centers. d) Lateral streams, extended almost 0.5 mm in *y*-direction, start to line up in the direction of flow.

**Fig 16:**
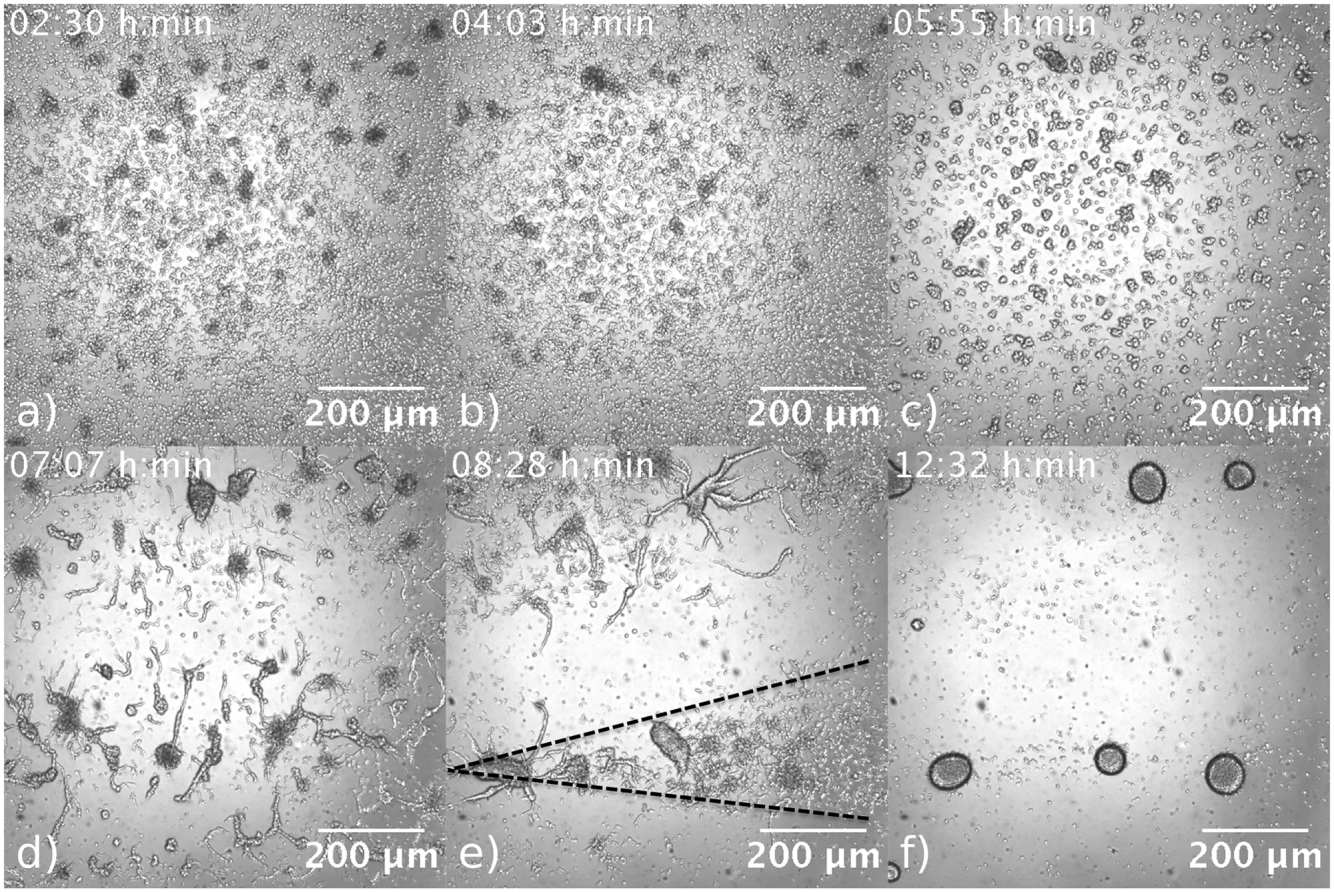
Aggregation process observed in a bright field microscope at *V*_*f*_ = 10 mm/min. Cone-shaped aggregation domains with long stream lines are visible in panel e).

To investigate aggregation dynamics of *D. discoideum* cells in the presence of flow, we performed experiments in which the flow is maintained well into the culmination phase of the life cycle. In our experiments, waves appear 3-6 hours after starvation. During this time, chemotactic cell movement is still weak [34] and the variations in cell density are not significant (compare Fig 15a and 15b). Later, 8 hours into starvation, cells form atypical aggregate patterns at high flow rates, as shown in Figs 15c and 16e. Similar to the experiments in Ref. [35], we observed cone-shaped long streams that existed in the downstream and lateral side of the centers. The lateral streams continue to line up in the direction of the imposed flow (see Fig 15d and supplemental S14 Video). Interestingly, the cells upstream the center do not sense any stimulus and aggregate randomly. The length of the long streams are about 4 mm, showing that the stimulus from the centers are extended over a long distance downstream so that only the cells directly downstream of the center will show any orientation. We also used bright field microscopy to closely look at the wave propagation and streaming process under flowing buffer. Snapshots of the cell distribution during the aggregation process are shown in Fig 16. In particular, the cone-shaped structures and long stream lines are well visible in Fig 16e and supplementary S15 Video.

## Discussion

We have found that colonies of *D. discoideum* initiate cAMP waves even if subjected to high flow rates (see [15] for results on lower flow rates). The speed of the waves along the channel is proportional to the imposed flow velocity, while both the velocity in the transversal direction and the wave period are independent of imposed flow velocity. The wave speed in transversal direction seems to be the same as in the spiral waves of *D. discoideum* populations in the absence of flow, where they move at 0.4 mm/min [32] [12] and the period found is consistent with the one of target patterns in previous studies of 5-7 min [32] / 5-8 min [36]. This is a strong indicator that period and wave speed are intrinsic characteristics of the system that allow for robust aggregation even under strenuous advecting flow conditions. It is interesting that *D. discoideum* cells aggregate even at high imposed flow velocities. The type of trailing edge we observe is similar to the ones previously observed in water flow experiments of Ref. [35].

The shape of the cAMP waves changes with higher imposed flow speeds, transitioning from a planar wave to a parabolic-shaped wave that becomes increasingly longer the higher the flow rate. At high buffer flow rates, the waves are extremely elongated and their observable portion moves mostly perpendicular to the flow. Indeed, we have observed waves that are extended over the entire length of the channel. These waves, just like parabolic-shaped waves, originate at the inlet either the top or the bottom of the channel. These observations are consistent with what we observed in simulations with a developmental path, where clusters of cells advanced on the path would fail to produce a wave unless they are located near the upstream boundary. If the waves are generated upstream bottom (or top) then the cAMP quickly gets advected downstream on a time scale that is much shorter than its degradation time, thus looking like a line of cAMP that moves upwards (downwards) at the usual wave propagation velocity (≈0.4 mm/min).

We also observed an increase of the wave intensity and width in the presence of flow both in experiments and simulations. We found wave widths of up to 3 mm, which are wider than the width of spiral waves in the undisturbed system, that have been reported around 0.3-1 mm [12] for the whole wave, and 0.7 mm at half height [32]. We understand the increase in width based on previous research showing that *D.discoideum* emits cAMP during a period of time of approximately *t*_*f*_ ≈ 3 − 4 min [37] / 1-3 min [32], thus in a first order approximation *d* = *v*_∥_ ⋅ *t*_*f*_ where *d* is wave thickness, *v*_∥_ wave propagation velocity along the flow, and *t*_*f*_ firing time. In other words, due to the flow, the cAMP produced covers a larger distance before the cell gets inactive. The increase in intensity when subjected to advection has also been observed in other reaction-diffusion systems, such as the Beluosov-Zhabotinsky reaction [38], while the increase in thickness has also been reported in numerical simulations of the FitzHugh-Nagumo model [39] and of autocatalytic fronts [40].

The wave initiation process is the most intriguing of the observed effects. We observed that for slow flow velocities the waves can start from anywhere in the system. For faster flows, we believe that the cAMP emitted by the centers is quickly advected away, and therefore do not create a supra-threshold perturbation in neighboring cells that would allow wave propagation. In the experiments, wave formation might be due to unbounded phosphodiesterase, since cells downstream would receive the enzyme secreted by the cells upstream, while the cells at the upper boundary receive a clean flow coming from the injected flow. Since this flow has an effective lower degradation, it allows for the creation of new wave pulses. More experiments using PDE deficient cells are necessary to confirm this hypothesis.

We tested the dynamical state of the cells by abruptly setting the flow velocity to zero and observing the response of the waves. In several experiments we find that the waves traveling along the channel move along it without flow as well. The waves moved at the speed of the imposed flow while the flow was applied and immediately slowed down to approximately the propagation velocity of spiral waves, as the flow was switched off. Furthermore, the width of the waves decreased, as the flow was switched off. This type of response is expected in excitable systems where a pulse has defined characteristics like speed and width given by the system to which the wave would return in the absence of flow. In contrast, in an oscillatory system a thicker wave would produce a synchronized (bulk) oscillation in that area, so even though the wave propagating would have a normal thickness, the area perturbed at the moment of the switch off would have a less organized behavior with areas of synchronized oscillations. We reproduced these results by switching off the flow in numerical simulations with developmental path. Even though the results depended weakly on at which point along the path the flow was stopped, they showed waves continuing to travel along the channel and some more disordered waves given by the oscillatory patches. Therefore a mixture of oscillatory and excitable cells managed to reproduce our experimental observations.

To summarize, we find that *D. discoideum* cells initiate defined cAMP waves even under the influence of strong advective flows up to *V*_*f*_ = 50 mm/min. We find that the waves change shape depending on the applied flow velocity, transitioning from planar waves at low speeds, to parabolic shaped waves, whose elongation increases with flow velocity. These wave shapes observed at high speeds were strongly dependent on the location of their initiation point. Since the cells move against cAMP gradients when aggregating, the shape of the cAMP waves have an important role on the aggregation process, in regards to this, it is noteworthy that even without the presence of wave centers such as target centers and spirals, the cells are still capable of aggregation. This also shows the capability of *D. discoideum* to signal even in extremely adverse conditions.

The wave speed in transversal direction *v*_⊥_ and the period of the waves *T* showed to be robust system characteristics, being constant for all studied flow speeds. The wave speed along the channel *v*_∥_ and the wave width *d*, however, scale linearly with *V*_*f*_. We compare these experimental results to numerical simulations of the system. While a two-component model was sufficient for low flow velocities, we find the extension to the three-component model necessary to reproduce the wave shape at higher flows, showing how fundamental the intracellular dynamic is to produce robust signaling.

Experiments conducted with switching off the advecting flow showed waves that travel along the system changing their velocity and width to recover their unperturbed characteristics, displaying a trigger wave behavior, characteristic of excitable systems.

The observed wave shapes were successfully reproduced using a developmental path which added desynchronization to the system, having some cells in an oscillatory state and some in an excitable one. The upstream cells more advanced in this path became the source of the downstream traveling waves. This scheme also reproduced successfully the flow switching off experiments. We expect our investigations to be crucial to understand signaling of *D. discoideum* cells in the presence of external flows.

## Supporting information

S1 File Shear stress calculations. (PDF)

S1 Fig Flow profile in the channel. Laminar flow profile inside the microfluidic channel in arbitrary units. a) Cut along the channel center (*y* = 0). b) Cut along half channel height(*z* = 0).
(EPS)

S1 Video Experiment without flow. The movie shows an experiment in the macro-channel without an imposed flow, so at *V*_*f*_ = 0. The three rows of this and later movies show in order: i) the original Dark-field images ii) the subtracted and band-pass filtered images and iii) the local phase extracted from the band-pass filtered images.
(MOV)

S2 Video Experiment with flow. Experiment with a low imposed flow of *V*_*f*_ = 0.5 mm/min showing a planar wave train.
(MOV)

S3 Video Experiment with flow. Experiment with an imposed flow of *V*_*f*_ = 5 mm/min showing parabolic waves.
(MOV)

S4 Video Experiment with flow. Experiment with an imposed flow of *V*_*f*_ = 10 mm/min showing strongly elongated parabolic waves.
(MOV)

S5 Video Experiment with flow. Experiment with an imposed flow of *V*_*f*_ = 15 mm/min showing extremely elongated parabolic waves.
(MOV)

S6 Video Simulations. Numerical simulation of the two-component model in the excitable regime with an imposed flow of *V*_*f*_ = 10 mm/min. Initial perturbation applied center upstream.
(MOV)

S7 Video Simulations. Numerical simulation of the three-component model in the excitable regime with an imposed flow of *V*_*f*_ = 10 mm/min. Initial perturbation applied center upstream.
(MOV)

S8 Video Simulations. Numerical simulation of the three-component model using a developmental path for the parameters *σ* and *k*_*e*_, with an imposed flow of *V*_*f*_ = 15 mm/min. Top: cAMP concentration. Bottom: State of the cells, gray for the stable state, purple for the oscillatory regime, and excitable regime in orange.
(MOV)

S9 Video Simulations. Numerical simulation of the three-component model using a developmental path for the parameters *σ* and *k*_*e*_, with an imposed flow of *V*_*f*_ = 15 mm/min. Top: cAMP concentration. Bottom: State of the cells, gray for the stable state, purple for the oscillatory regime, and excitable regime in orange.
(MOV)

S10 Video On-off flow experiment. Experiment which had no flow initially, at *t*_1_ = 4 h 27 min a flow of *V*_*f*_ = 1 mm/min was switched on. The flow was switched off again at *t*_3_ = 5 h 21 min.
(MOV)

S11 Video Simulations. Numerical simulation of the three-component model using a developmental path for the parameters *σ* and *k*_*e*_, with an imposed flow of *V*_*f*_ = 1 mm/min until *t* = 250 min, afterwards the flow is off, *V*_*f*_ = 0 mm/min. Top: cAMP concentration. Bottom: State of the cells, gray for the stable state, purple for the oscillatory regime, and excitable regime in orange.
(MOV)

S12 Video Jump-in-flow experiment. Experiment which had no flow initially. At 3 h 46 min a flow of *V*_*f*_ = 1 mm/min was switched on. Next the flow was increased first to *V*_*f*_ = 2 mm/min at 4 h 09 min, then to *V*_*f*_ = 3 mm/min at 4 h 24 min. Finally, the speed was increased to *V*_*f*_ = 4 mm/min at 4 h 48 min. At 5 h 51 min the flow was reduced to *V*_*f*_ = 1 mm/min again.
(MOV)

S13 Video Simulations. Numerical simulation of the three-component model using a developmental path for the parameters *σ* and *k*_*e*_, with an imposed flow of *V*_*f*_ = 1 mm/min until *t* = 220 min, afterwards the flow is increased to *V*_*f*_ = 2 mm/min and at *t* = 250 min increased to *V*_*f*_ = 3 mm/min. Top: cAMP concentration. Bottom: State of the cells, gray for the stable state, purple for the oscillatory regime, and excitable regime in orange.
(MOV)

S14 Video Aggregation experiment. Experiment with *V*_*f*_ = 10 mm/min showing original Dark-field images for a longer time to show the aggregation phase of the cells.
(MOV)

S15 Video Bright-field experiment. Bright-field microscopy experiment showing the aggregation in a channel with *V*_*f*_ = 10 mm/min (4X magnification).
(MOV)
