# Supplementary material for: Influence of fast advective flows on pattern formation of Dictyostelium discoideum
Source: PLoS One. 2018 Mar 28;13(3):e0194859. doi: 10.1371/journal.pone.0194859 (PMC5874059; doi:10.1371/journal.pone.0194859)
Supplement: S1 File — (PDF) [file pone.0194859.s001.pdf]

Shear stress

To calculate the shear stress acting on the cells in the microfluidic channel, we solve the incompressible Navier-Stokes equation in 3D. The channel has a rectangular geometry ($x \in [0, L]$, $y \in [-c, c]$, $z \in [-b, b]$) and we use zero velocity boundary conditions at the boundaries of the channel cross section ($u(y = \pm c) = 0$ and $u(z = \pm b) = 0$),

$$\rho \frac{Du}{Dt} = \rho g - \nabla p + \mu \nabla^2 u.$$

Here μ denotes fluid viscosity, ρ fluid density, p pressure, and u fluid velocity. We solve using variable separation and Fourier series, and find an expression for the fluid velocity u

$$u = \frac{G(b^2 - z^2)}{2\mu} + \sum_{n \text{ odd}} A_n \cosh\left(\frac{n\pi}{2b} y\right) \cos\left(\frac{n\pi}{2b} z\right), \qquad \text{with}$$
$$A_n = -\frac{16 G b^2}{\mu \pi^3 n^3} \frac{\sin\left(\frac{n\pi}{2}\right)}{\cosh\left(\frac{n\pi c}{2b}\right)}.$$

Where G denotes the pressure drop in the channel. For a detailed derivation of the velocity please see [1].

We now calculate the wall shear stress from the velocity profile, using the expression

$$\tau = \mu \partial_z u|_{z=-c},$$

and the velocity space derivative

$$\partial_z u = -\frac{Gz}{\mu} - \frac{n\pi}{2b} \sum_{n \text{ odd}} A_n \cosh\left(\frac{n\pi}{2b} y\right) \sin\left(\frac{n\pi}{2b} z\right).$$

Considering we do not have access to the pressure drop G across the channel, but only the mean flow Q we find an expression for the mean flow by integrating over the channel cross section,

$$Q = \frac{G b^3 c}{\mu} \left[\frac{4}{3} - \frac{8b}{c} \sum_{n \text{ odd}} \left(\frac{2}{n\pi}\right)^5 \tanh\left(\frac{n\pi c}{2b}\right)\right],$$

thus we obtain the pressure drop G

$$G = \frac{Q\mu}{b^3 c} \left[\frac{4}{3} - \frac{8b}{c} \sum_{n \text{ odd}} \left(\frac{2}{n\pi}\right)^5 \tanh\left(\frac{n\pi c}{2b}\right)\right]^{-1}.$$

We can simplify G by using the values for c and b. Note also that since $\pi c/(2b) \approx 31.4$ the hyperbolic tangent becomes $tanh(\pi c/(2b)) \approx 1$, this leads to

$$G = \frac{Q\mu}{b^3 c} \times \left[\frac{4}{3} - \epsilon\right]^{-1} \qquad \text{with}$$
$$\epsilon = \frac{8b}{c} \sum_{n \text{ odd}} \left(\frac{2}{n\pi}\right)^5 \tanh\left(\frac{n\pi c}{2b}\right) \approx 4.2 \times 10^{-2}.$$

V_f $[\frac{mm}{min}]$	τ [Pa]
1	0.000918
5	0.004590
10	0.009180
15	0.013771
20	0.018361
30	0.027541
50	0.045902

Table 1. Shear stress τ calculated as a function of imposed flow velocity V_f, using $\mu = 8.9 \cdot 10^{-4}$ Pa s, the dynamic viscosity of water and the channel dimensions $c = 1$ mm and $b = 0.05$ mm.

Then we can approximate this for small ϵ

$$G \approx \frac{Q\mu}{b^3 c}\left[\frac{3}{4} + \left(\frac{3}{4}\right)^2 \epsilon\right].$$

Since we want to find the maximum stress, we choose y to be at the middle of the channel ($y = 0$). Thus, we can simplify τ as well, since $\cosh(n\pi y/2b) = 1$, and $A_n \approx 0$ $\forall n \in \mathbb{N}$,

$$\tau_{y=0} = -Gz = -\frac{Q\mu z}{b^3 c} \times \left[\frac{3}{4} + \left(\frac{3}{4}\right)^2 \epsilon\right].$$

We can now calculate the shear stress for the imposed mean flow velocities used in our experiments. An overview is shown in Table 1.
Thus, the shear stresses are well below the critical value for the onset of mechanosensivity 0.7 Pa or even detachment 2.6 Pa [2].

References

1. Vidal-Henriquez E, Zykov V, Bodenschatz E, Gholami A. Convective instability and boundary driven oscillations in a reaction-diffusion-advection model. Chaos: An Interdisciplinary Journal of Nonlinear Science. 2017;27(10):103110. doi:10.1063/1.4986153.

2. Décavé E, Rieu D, Dalous J, Fache S, Bréchet Y, Fourcade B, et al. Shear flow-induced motility of Dictyostelium discoideum cells on solid substrate. Journal of cell science. 2003;116(21):4331–4343. doi:10.1242/jcs.00726.
